# The effect on the small bowel of 5-FU and oxaliplatin in combination with radiation using a microcolony survival assay

**DOI:** 10.1186/1748-717X-4-61

**Published:** 2009-12-09

**Authors:** Adalsteinn Gunnlaugsson, Per Nilsson, Elisabeth Kjellén, Anders Johnsson

**Affiliations:** 1 Department of Oncology, Lund University Hospital, Lund Univeristy, Lund, Sweden; 2 Department of Radiation Physics, Lund University Hospital, Lund Univeristy, Lund, Sweden; 3 Department of Radiation Sciences, Oncology, Umeå University, Umeå, Sweden

## Abstract

**Background:**

In locally advanced rectal cancer, 5-Fluorouracil (5-FU)-based chemoradiation is the standard treatment. The main acute toxicity of this treatment is enteritis. Due to its potential radiosensitizing properties, oxaliplatin has recently been incorporated in many clinical chemoradiation protocols. The aim of this study was to investigate to what extent 5-FU and oxaliplatin influence the radiation (RT) induced small bowel mucosal damage when given in conjunction with single or split dose RT.

**Methods:**

Immune competent balb-c mice were treated with varying doses of 5-FU, oxaliplatin (given intraperitoneally) and total body RT, alone or in different combinations in a series of experiments. The small bowel damage was studied by a microcolony survival assay. The treatment effect was evaluated using the inverse of the slope (D_0_) of the exponential part of the dose-response curve.

**Results:**

In two separate experiments the dose-response relations were determined for single doses of RT alone, yielding D_0 _values of 2.79 Gy (95% CI: 2.65 - 2.95) and 2.98 Gy (2.66 - 3.39), for doses in the intervals of 5-17 Gy and 5-10 Gy, respectively. Equitoxic low doses (IC5) of the two drugs in combination with RT caused a decrease in jejunal crypt count with significantly lower D_0_: 2.30 Gy (2.10 - 2.56) for RT+5-FU and 2.27 Gy (2.08 - 2.49) for RT+oxaliplatin. Adding both drugs to RT did not further decrease D_0_: 2.28 Gy (1.97 - 2.71) for RT+5-FU+oxaliplatin. A clearly higher crypt survival was noted for split course radiation (3 × 2.5 Gy) compared to a single fraction of 7.5 Gy. The same difference was seen when 5-FU and/or oxaliplatin were added.

**Conclusion:**

Combining 5-FU or oxaliplatin with RT lead to an increase in mucosal damage as compared to RT alone in our experimental setting. No additional reduction of jejunal crypt counts was noted when both drugs were combined with single dose RT. The higher crypt survival with split dose radiation indicates a substantial recovery between radiation fractions. This mucosal-sparing effect achieved by fractionation was maintained also when chemotherapy was added.

## Background

Surgery is the cornerstone of curative therapy for colorectal cancer. In locally advanced cases, radiotherapy is used preoperatively to shrink the tumor in order to facilitate a curative resection. An improved effect has been shown when radiation is combined with the chemotherapeutic agent, 5-fluorouracil (5-FU) [1-3]. This has made 5-FU-based chemoradiation a standard treatment for locally advanced rectal cancer. Another drug, oxaliplatin, has become widely used in adjuvant [4], as well as palliative [5,6] treatment of colorectal cancer. Several phase II studies indicate good efficacy when oxaliplatin was combined with 5-FU or oral fluoropyrimidines such as capecitabine and radiotherapy [7-11]. Preclinical studies have demonstrated that both 5-FU and oxaliplatin have radiosensitizing properties on tumor cell lines *in vitro *[12,13], while the additional effect of oxaliplatin *in vivo *is more uncertain [13,14].

The main dose-limiting acute side effect during abdominal radiation is enteritis. Randomized studies have shown that combined treatment with radiation and 5-FU increases the risk of diarrhea as compared to radiotherapy alone [2]. There are no published results from randomized trials on whether the addition of oxaliplatin further increases gastrointestinal toxicity, but the combination of 5-FU (or capecitabine), oxaliplatin and radiotherapy has lead to 12-37% grade 3+ enteritis in phase II trials [7-11].

The regeneration of the bowel mucosa is dependent on its clonogenic stem cells which are located in the small bowel crypts. Therefore, the survival of these clonogens is likely to be a decisive factor in the repair of the bowel after cytotoxic therapy. The pioneering work of Withers and Elkind presented in 1970 has given us the opportunity to study this in the mouse intestinal mucosa [15]. Development of radiation enteritis is thought to be mediated through a toxic effect on these mucosal stem cells. The aim of this study was to study the bowel damage caused by radiation, 5-FU or oxaliplatin as well as combinations thereof by using a microcolony survival assay and comparing the difference between single and split dose radiotherapy.

## Methods

### Mice

Immune competent balb-c mice were used. The mice were treated at the age of six to seven weeks and were housed in well-ventilated lucite boxes with food and water ad libitum. The study was approved by the Malmö-Lund animal ethics committee.

### Irradiation

Total body irradiation was administered with a 6 MV photon beam from a medical linear accelerator at a dose rate of 3 Gy/min. The animals were treated five at a time in a lucite box, specially designed for obtaining a homogenous total body dose (within ± 5%) and for gentle fixation of the animals. Control animals were sham irradiated. The time of radiation was defined as time point 0. The radiotherapy was given as a single fraction (0, 2.5, 5, 7.5, 10, 14, or 17 Gy) or as a split dose treatment with 2.5 Gy fractions delivered two or three times with six-hour intervals. Five mice were treated at each radiation dose level.

### 5-FU and oxaliplatin

Both drugs were administered intraperitoneally. Single doses of 5-FU (Mayne Pharma) 0-200 mg/kg and of oxaliplatin (Mayne Pharma) 0-10 mg/kg were administered alone or in combination with radiotherapy. When combined, 5-FU was injected immediately followed by the administration of oxaliplatin one hour prior to radiotherapy. Control animals were given saline injection instead of chemotherapy. For each different dose level, five mice were treated.

### Microcolony assay

A microcolony survival assay [15] was used to analyze crypt survival after treatment. Three days after radiotherapy or chemotherapy, the mice were killed by cervical dislocation and a 10 cm section of the jejunum was collected, stretched and pinned to a cork plate to ease histological preparation. Tissues were fixed immediately in 4% formalin with phosphate buffer and 10 transverse sections of jejunum from each mouse were prepared and stained with hematoxylin and eosin (H&E). These transverse sections were analyzed microscopically, and a surviving colony was defined as one demonstrating the presence of ten or more well-stained cells in the section. The slides were analyzed by one observer (A.G.) in a blinded fashion.

### Data analysis

The number of regenerating crypts/circumference was counted for each section from each treated (t) or untreated (control, c) animal. The surviving crypt fraction was thus t/c and the proportion of crypts destroyed/transverse section was 1-t/c. Since ten transverse sections were obtained from each mouse and five mice were used for each treatment dose, each data point was composed of a maximum of 50 observations. A linear regression was done to estimate D_0_, i.e., the inverse of the slope of the exponential part of the survival curve, for radiotherapy alone and for the combinations of 5-FU and/or oxaliplatin with radiotherapy. The Mann-Whitney test was used to compare crypt survival after single versus split-dose radiotherapy. All tests were two-sided and p-values < 0.05 were considered statistically significant.

### Experimental design

Four sets of experiments were performed as follows:

1. The effect of radiation alone was studied at single doses ranging from 0 - 17 Gy, with the purpose of describing the dose-response relations in our experimental setting and finding appropriate doses for the combined treatment (step 3).

2. The effect of each drug alone, at different doses, was studied to find suitable doses which produced moderate and equal intestinal damage. These doses were then combined with radiation in the next step.

3. Single radiation fractions, ranging from 0 - 10 Gy, were given alone, or in combination with equitoxic single doses of 5-FU, oxaliplatin, or both drugs.

4. The same single doses of 5-FU and oxaliplatin were combined with split dose radiation.

## Results

A total of 265 mice were used in the study. Nineteen died before histological analysis: radiation alone (n = 4), 5-FU alone (n = 1), oxaliplatin alone (n = 6), radiation + 5-FU (n = 2), radiation + oxaliplatin (n = 3), radiation + 5-FU + oxaliplatin (n = 2) and 5-FU + oxaliplatin (n = 1) and thus 246 mice were available for crypt analysis. A total of 2206 transverse sections (mean 45 sections per data point) were available for analysis.

### Radiotherapy

**Figure 1:**
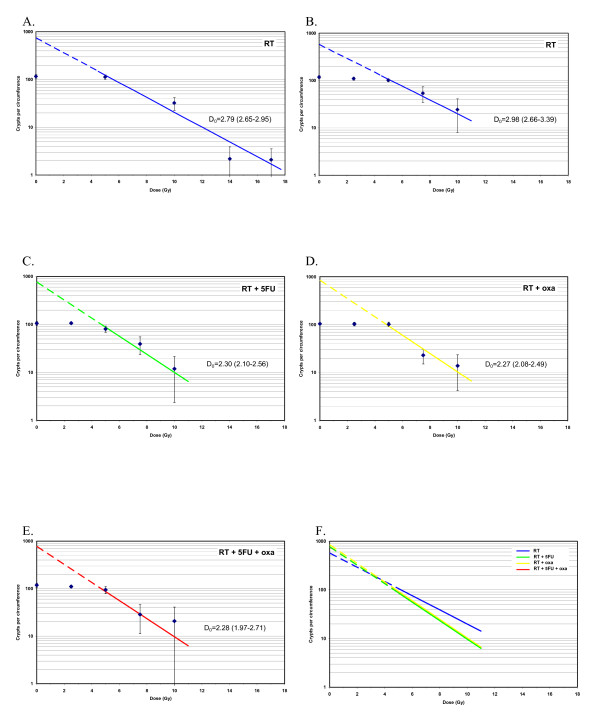
**The number of surviving crypts per circumference and D_0_with 95% confidence interval after A**. Radiotherapy (1^st ^experiment, 0-17 Gy), B. Radiotherapy (2^nd ^experiment, 0-10 Gy), C. 5-FU + radiotherapy (0-10 Gy), D. Oxaliplatin + radiotherapy (0-10 Gy) and E. 5-FU + oxaliplatin + radiotherapy (0-10 Gy). F. Survival curves for all treatment combinations above with separate data points removed for clarity. Each data point represents the mean in each group and the error bars 1 SD. Oxaliplatin dose: 6 mg/kg, 5-FU dose: 50 mg/kg.

The first radiation series with doses of 0, 5, 7.5, 10, 14 and 17 Gy is visualized in Fig. 1A. The surviving crypts per circumference decreased with increasing radiation doses showing a dose-response relationship. D_0 _was calculated using data points for doses from 5 - 17 Gy where the dose-effect curve was considered exponential. In this first experiment we found a D_0 _of 2.79 Gy (95% CI: 2.65 - 2.95). The highest radiation doses of 14 - 17 Gy caused a near complete eradication of jejunal crypts (Fig. 1A). When planning the chemoradiation experiment, we assumed that radiation doses of 14-17 Gy plus chemotherapy also would lead to zero crypt count, and the highest radiation doses were therefore omitted in the studies of combined treatment. The result from the radiation alone experiment with doses in the 0-10 Gy range is depicted in Fig. 1B, demonstrating a D_0 _of 2.98 Gy (95% CI: 2.66 - 3.39).

### Chemotherapy

**Figure 2:**
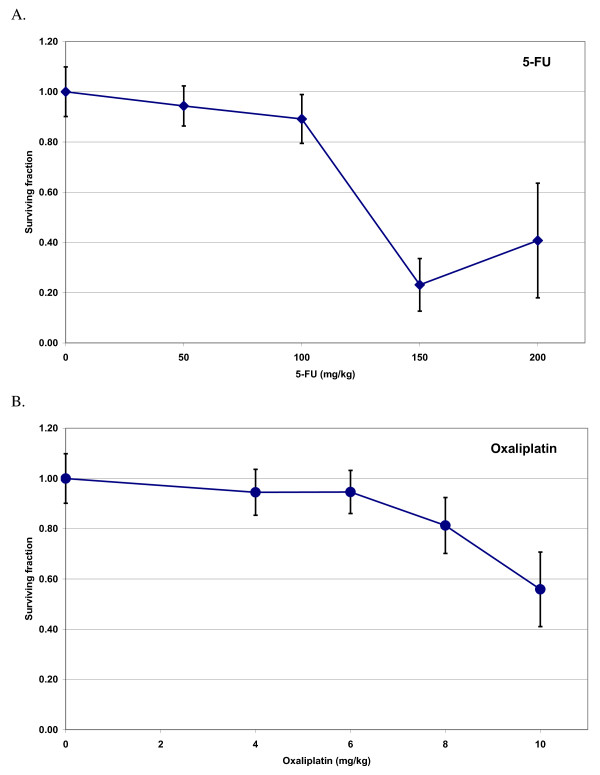
**The surviving fraction of crypts per circumference as a function of chemotherapy dose**. A. 5-Fluorouracil (5-FU), B. Oxaliplatin. Each data point stands for the mean of each group and error bars represent +/- 1 SD.

5-FU administration decreased the surviving fraction of crypts per circumference up to a dose of 150 mg/kg (Fig. 2A), followed by a further, slight increase in the mean crypt level at the highest 5-FU dose (200 mg/kg). In order to rule out a methodological error as an explanation for this finding, these two 5-FU doses were reevaluated in a separate experiment, which confirmed our original result, although again with large error bars at this dose level. Increasing doses of oxaliplatin resulted in an essentially linear decrease in the number of surviving crypts in the dose range from 6 to 10 mg/kg (Fig. 2B).

### Chemoradiation - single fraction radiation

The experiment described above (Fig. 2A-B) showed that a 5-FU dose of 50 mg/kg and an oxaliplatin dose of 6 mg/kg each had a modest and equal effect on the intestinal crypts, and these doses were chosen for combination with radiation.

The addition of 5-FU (Fig. 1C) or oxaliplatin (Fig. 1D) to radiotherapy significantly decreased the number of surviving crypts per circumference as compared to radiation alone. The D_0 _for radiation decreased from 2.98 Gy to 2.30 Gy (p = 0.001) and 2.27 Gy (p = 0.0003) when 5-FU and oxaliplatin were added respectively.

The combination of both oxaliplatin and 5-FU with radiotherapy did not lead to any further decrease in D_0 _as compared to the addition of each drug alone (Fig. 1E-F).

### Split-dose radiotherapy

Splitting the radiation dose into two or three fractions lead to significantly more surviving crypts per circumference as compared to the same total dose given in one fraction (Table 1), with the largest difference noted for 3 × 2.5 Gy compared to 7.5 Gy as a single treatment. Also when adding chemotherapy, there were clearly more surviving crypts with fractionated radiation compared to chemotherapy plus the same radiation dose given as a single dose.

**Table 1:** Comparison of single versus split dose irradiation with or without concomitant chemotherapy

Treatment	Crypt count (95% CI)	p-value
5 Gy	101 (99-103)	< 0.0001
	
2 × 2.5 Gy	114 (111-116)	

5 Gy + 5-FU	80 (66-95)	< 0.0001
	
2 × 2.5 Gy + 5-FU	102 (94-111)	

5 Gy + oxa	101 (94-108)	0.5
	
2 × 2.5 Gy + oxa	102 (91-112)	

5 Gy + 5-FU + oxa	88 (77-98)	0.007
	
2 × 2.5 Gy + 5-FU + oxa	93 (75-111)	

7.5 Gy	54 (29-80)	< 0.0001
	
3 × 2.5 Gy	99 (93-107)	

7.5 Gy + 5-FU	39 (20-58)	< 0.0001
	
3 × 2.5 Gy + 5-FU	95 (81-110)	

7.5 Gy + oxa	23 (15-31)	< 0.0001
	
3 × 2.5 Gy + oxa	91 (84-99)	

7.5 Gy + 5-FU + oxa	29 (0-57)	< 0.0001
	
3 × 2.5 Gy + 5-FU + oxa	97 (91-103)	

5-FU dose = 50 mg/kg, oxaliplatin dose = 6 mg/kg

Abbreviations: oxa = oxaliplatin

## Discussion

This is to our knowledge the first study on chemoradiation-induced bowel mucosal damage in mice including oxaliplatin. A dose relationship was confirmed between radiation dose and crypt survival (Fig. 1A-B). Adding 5-FU or oxaliplatin lead to a significant increase in jejunal crypt damage, in terms of decreased D_0_, compared to radiation alone (Fig. 1C-D). The co-administration of both drugs did not further increase radiation induced mucosal damage (Fig. 1E). Fractionated radiation caused less mucosal damage than the same total dose given as a single fraction. This damage-sparing effect by fractionating the radiation was retained also when chemotherapy was added (Table 1).

The initial part of our study aimed at determining the mucosal injury caused by radiation alone. In the two series using radiation doses up to 17 Gy and 10 Gy, we found D_0 _values of 2.79 and 2.98, respectively, which is higher than usually reported in the literature (typically in the range 1-1.5 [16,17]). In those studies D_0 _was calculated at radiation doses ranging from around 9 - 14 Gy [17], compared to 5 Gy and higher in the present study. Using only data points from 7.5 or 10 Gy and higher did not significantly decrease the D_0 _in our study (data not shown). One possible explanation for the inter-study discrepancies could be variations in inherent radiosensitivity between different mouse strains [18]. Since radiation doses of 14 to 17 Gy lead to a near complete eradication of jejunal crypts, we chose to use only doses up to 10 Gy in the combined chemoradiation experiments. Besides, the aim of our study was not to determine the absolute D_0 _values but rather to investigate the relative impact on jejunal damage by adding 5-FU and oxaliplatin to radiation.

5-FU has been subjected to several previous studies using murine models. The doses chosen for our experiments have shown antitumoral efficacy with reasonable toxicity in these studies [19]. We found that doses above 100 mg/kg resulted in surviving fractions between 20 and 40% (Fig. 2A), which indicates a stronger cytotoxic effect than previous studies using the microcolony assay for 5-FU [20]. One explanation for this lower clonogenic cell recovery may be the slightly shorter time span from treatment to analysis compared to other similar studies [20].

Regarding oxaliplatin, no previous studies have been published on its effect on jejunal clonogenic crypt survival, neither alone nor in combination with radiotherapy. The oxaliplatin doses tested, from 4 to 10 mg/kg, have previously been used in combination with radiotherapy in xenografted mice and have shown antitumoral effect and limited general toxicity [14]. Our study showed a slight to moderate drop in jejunal crypt surviving fraction within that dose range (Fig. 2B), when administering oxaliplatin alone.

In the chemoradiation experiments we used chemotherapy doses that caused a low degree of mucosal damage on their own. This principle is often applied also in the clinical setting. Despite these low doses we saw a significant reduction of the D_0 _values by adding either of the two drugs to radiation compared to radiation alone, which indicates that both 5-FU and oxaliplatin may potentiate radiation-induced mucosal damage. However, there was no additional jejunal injury when both drugs were added to radiation.

When treating patients with colorectal cancer, radiation doses higher than 5 Gy per fraction are usually not used, especially not in combination with chemotherapy. To better mimic the clinical situation, we investigated the effect of fractionated radiation. Compared with 5 and 7.5 Gy as a single dose, 2 and 3 × 2.5 Gy resulted in considerably less jejunal damage (Table 1), indicating a substantial cellular recovery during the 6 h time span between radiation fractions. The fact that there was no significant reduction of crypt survival when chemotherapy was added to split dose radiation, indicates that neither 5-FU nor oxaliplatin seem to abolish the mucosal-sparing effect achieved by fractionating the radiation. To elucidate this further a larger study with graded fraction doses is needed where alpha/beta values for these treatments can be calculated.

How do these results correlate to the clinical experience? 5-FU is known to cause mucositis, which can involve the intestines and cause enteritis. Depending on the schedule of administration, the frequencies of grade 3-4 diarrhea were 3% and 7% for infusional and bolus regimens, respectively, in a randomized trial [21].

For oxaliplatin as single treatment, a grade 3-4 diarrhea frequency of 6% has been reported [22]. For radiotherapy, the relationship between toxicity and radiation dose is well known with the grade of diarrhea also correlated with the irradiated volume of the small bowel [23,24]. Addition of 5-FU to radiotherapy has been shown in two randomized trials to increase the risk of enteritis [2,3]. Thus the radiosensitization observed for 5-FU and oxaliplatin alone in the present study is in concordance with clinical experience.

Whether the combination of both drugs synergistically leads to considerable increase in bowel toxicity is not well known, since no randomized trials on this issue have been published yet. Several phase I and II studies on oxaliplatin-based chemoradiation have been performed [7-11], yielding grade 3-4 diarrhea that seems slightly higher (12-37%) than in protocols using only 5-FU or capecitabine together with radiotherapy [2,3,25,26]. In our experimental setting there were no signs of additional radiosensitization when oxaliplatin was added to radiation and 5-FU with identical D_0 _values (Fig. 1E-F) and no detrimental effect on recovery (Table 1). Results from ongoing randomized trials will show whether this is true also in the clinical setting. One cannot exclude that using higher or multiple chemotherapy doses, more radiation fractions or different mouse strains would have led to a further decrease in D_0 _when combining both drugs with radiotherapy. The basis for this clonogenic assay is that the regeneration of the bowel mucosa is dependent on its clonogenic stem cells. Therefore, the survival of these clonogens is likely to be a decisive factor in the repair of the bowel after cytotoxic therapy. However, it is possible that other factors, such as inflammation and bacterial disturbances, also may add to chemoradiation-induced enteritis in the clinical situation.

## Conclusion

In conclusion, the addition of 5-FU or oxaliplatin to radiotherapy lead to a similar decrease in jejunal crypt survival for both drugs. Adding the drugs together with radiation did not further increase the mucosal damage in this experimental setting.

## Competing interests

The authors declare that they have no competing interests.

## Authors' contributions

All authors have contributed to the study design, data analysis, manuscript drafting and revising and given final approval of the version to be published.
